# Dysglycaemia, Inflammation and Psychosis: Findings From the UK ALSPAC Birth Cohort

**DOI:** 10.1093/schbul/sby040

**Published:** 2018-04-09

**Authors:** Benjamin Ian Perry, Rachel Upthegrove, Andrew Thompson, Steven Marwaha, Stanley Zammit, Swaran Preet Singh, Golam Khandaker

**Affiliations:** 1 Department of Psychiatry, Coventry and Warwickshire Partnership NHS Trust, Coventry, UK; 2 Unit of Mental Health and Wellbeing, University of Warwick, Coventry, UK; 3 Insitute for Mental Health, University of Birmingham, Birmingham, UK; 4 Department of Psychiatry, Birmingham and Solihull Mental Health Foundation Trust, Birmingham, UK; 5 Centre for Academic Mental Health, School of Social and Community Medicine, University of Bristol, Bristol, UK; 6 Institute of Psychological Medicine and Clinical Neurosciences, Medical Research Council Centre for Neuropsychiatric Genetics and Genomics, Cardiff University, Cardiff, UK; 7 Department of Psychiatry, University of Cambridge, Cambridge, UK; 8 National Institute for Health Research Cambridge Biomedical Research Centre, Cambridge, UK; 9 Department of Psychiatry, Cambridgeshire and Peterborough National Health Service Foundation Trust, Cambridge, UK

**Keywords:** dysglycaemia, insulin resistance, psychosis, risk, schizophrenia, inflammation, ALSPAC

## Abstract

**Background:**

Psychosis is associated with both dysglycaemia and low-grade inflammation, but population-based studies investigating the interplay between these factors are scarce.

**Aims:**

(1) To explore the direction of association between markers of dysglycaemia, inflammation and psychotic experiences (PEs); and (2) To explore whether dysglycaemia moderates and/or mediates the association between inflammation and PEs.

**Method:**

Data from the Avon Longitudinal Study of Parents and Children (ALSPAC) birth cohort were modeled using logistic and linear regression to examine cross-sectional and longitudinal associations between markers of dysglycaemia (ages 9 and 18), interleukin-6 (IL-6) (age 9), and PEs (ages 12 and 18). We tested for an interaction between dysglycaemia and IL-6 on risk of PEs at age 18, and tested whether dysglycaemia mediated the relationship between IL-6 and PEs.

**Results:**

Based on 2627 participants, at age 18, insulin resistance (IR) was associated with PEs (adjusted OR = 2.32; 95% CI, 1.37–3.97). IR was associated with IL-6 both cross-sectionally and longitudinally. Interaction analyses under a multiplicative model showed that IR moderated the association between IL-6 at age 9 and PEs at age 18 (adjusted OR for interaction term = 2.18; 95% C.I., 1.06–4.49). Mediation analysis did not support a model of IR mediating the relationship between IL-6 and PEs.

**Implications:**

IR is associated with PEs in young people even before the onset of clinical psychosis. Metabolic alterations may interact with childhood inflammation to increase risk of PEs. The findings have implications for clinical practice and future research.

## Introduction

It is over 100 years since Henry Maudsley first reported an association between dysglycaemia and psychotic illness^1^ (then *dementia praecox*), decades before the advent of antipsychotic medication. Further corroborative reports^2–4^ in the pre-antipsychotic era supported this association, but these have been often overlooked after the detrimental effects of antipsychotics on metabolic indices were discovered.^5,6^ Recent meta-analyses have found sensitive measures of dysglycaemia (insulin resistance [IR], impaired glucose tolerance [IGT] and fasting insulin [FI]) to be associated with first-episode psychosis (FEP), independent of antipsychotic use and sociological/anthropomorphic factors,^7,8^ suggesting that the association between dysglycaemia and psychosis extends beyond the effects of antipsychotic medication, reduced access to healthcare and lifestyle factors. However, most research in the field has been cross-sectional, with little exploration of the longitudinal association between dysglycaemia and psychosis risk. Glycaemia exists on a continuum, as do psychoses.^9^ Examination of the relationship between dysglycaemia and psychotic experiences (PEs) in young people may provide evidence for an association unbiased by antipsychotic medication, chronicity of illness, physical comorbidities, and smoking, alcohol and other lifestyle factors.

A potential role of the immune system in the pathogenesis of psychosis has been proposed^10^ and low-grade systemic inflammation may link dysglycaemia and psychosis in a subgroup of patients. Evidence indicates that inflammatory cytokines such as interleukin-6 (IL-6) and tumor necrosis factor alpha (TNF-α) contribute to the pathogenesis of glycaemic dysfunction.^11–13^ Additionally, elevated concentrations of C-reactive protein (CRP), IL-6, and other inflammatory markers are also elevated in patients with established schizophrenia^14^ and unmedicated FEP,^15^ and research from large birth cohorts has reported that increased levels of IL-6 and CRP during childhood and adolescence are associated with subsequent PEs and a diagnosis of schizophrenia.^16,17^ A functional variant in the IL-6R gene, which is known to dampen inflammation, is protective for psychosis,^18^ and a recent Mendelian randomization study has shown a causal link between IL-6 and psychosis.^19^ Dysglycaemia and inflammation may be associated with psychosis in a mediating relationship (inflammation leading to dysglycaemia leading to risk of psychosis), or a moderating relationship (inflammation associated with psychosis only in a pathway that also results in dysglycaemia).

We aimed to examine cross-sectional and longitudinal associations between measures of dysglycaemia, inflammation, and risk of PEs/psychotic disorder in a large population birth cohort from the United Kingdom. We hypothesized that dysglycaemia would be associated with PEs/psychotic disorder, at younger ages than has been previously examined, after adjustments. We also aimed to test for mediation and/or moderation between inflammation and dysglycaemia for risk of PEs/psychotic disorder. Finally, we also examined the broader “metabolic syndrome” (MS) as an exposure, since there is research suggesting the intrinsic presence of MS in FEP.^20^

## Methods

### Description of Cohort and Sample Selection

The Avon Longitudinal Study of Parents and Children (ALSPAC) birth cohort comprises 14,062 live births from mothers residing in (former) Avon County, southwest England, with expected dates of delivery between April 1991 and December 1992 (http://www.bristol.ac.uk/alspac/). Full cohort details appear elsewhere.^21^ A fully searchable data dictionary is available at (http://www.bris.ac.uk/alspac/researchers/data-access/data-dictionary/).

Our sample frame consisted of participants that had data on all relevant variables (outcome measures, exposures, and adjustment variables). Data for cross-sectional analysis between dysglycaemia and psychotic symptoms/psychotic disorder at age 18 were available from 2627 participants. Data for longitudinal analysis of markers of dysglycaemia at age 9 years and psychotic symptoms at age 12/18 years were available from 387 participants. The study received ethics approval from the ALSPAC Law and Ethics Committee and local research ethics committees. All participants provided written informed consent.

## Laboratory Measures

### Markers of Dysglycaemia (Ages 9 and 18 y)

We used the biochemical measurements of fasting plasma glucose (FPG), FI (ages 9 and 18 y), and 2-hour glucose tolerance (2hrGT) (age 9 y). Age 9 glycaemic data was derived from a smaller sub-study.^22^ Fasting samples were taken at 9 AM after a 10-hour fast (only water allowed during the fasting period). The 2hrGT test was obtained following the above procedure, with the addition of a 75 g oral bolus of sugary syrup at 9 AM, with blood sampled 2 hours later.

IR was calculated from FPG and FI by using the computerized, updated version of the Homeostasis Model Assessment for IR (HOMA_2_).^23^ The algorithm generates a precise measurement of IR taking into account variations in hepatic and peripheral glucose resistance, increases in the insulin secretion curve for plasma glucose concentrations above 10 mmol/l and the contribution of circulating proinsulin.^23^

### Cut-off for IR and IGT

In addition to a continuous HOMA_2_ variable, we used a binary “clinical IR” variable. There is no consensus-agreed cut-off for clinical IR in the literature using HOMA_2_,^24–27^ since levels can be affected by multiple factors, including the study population.^28^ Proposed cut-offs range from >1.0^26^ through to 1.80^25^, with additional cut-offs suggested in-between these values.^24,27^ In lieu of an agreed cut-off, we chose to use the higher of the proposed cut-offs (>1.79), to maximize the specificity of our results. However, as a sensitivity analysis, we used the 75th centiles of the study populations (at ages 9 and 18 y) to define clinical IR (1.30 and 1.50, respectively) for each analysis.

In addition to the continuous 2hrGT variable, a binary variable was derived from the consensus-accepted clinical cut-off for IGT (>7.8 mmol/l).^29^

### MS at Age 18 Years

We constructed a binary MS variable using data available at age 18 years. There are numerous definitions of the MS, however, since our paper focuses on glycaemic abnormality, we used The World Health Organization (WHO) criteria,^30^ which emphasizes glycaemic abnormality. The WHO criteria specifies either T2DM or IR/IGT, plus 2 of either raised blood pressure (>140/90), dyslipidaemia (triglycerides [TG] >1.695 mmol/liter and high-density lipoprotein (HDL) < 0.9 mmol/l), central obesity (waist:hip ratio >0.90 or body mass index [BMI] >30 kg/m^2^), and microalbuminuria (urinary albumin excretion ratio >20 µg/min or albumin:creatinine ratio > 30 mg/mmol). From the ALSPAC dataset, we were able to obtain, in addition to the measures of glycaemia outlined above, TG, HDL, and BMI. Blood samples for HDL and TG were collected from fasting participants. As data were unavailable for some of the WHO criteria, we defined MS as presence of IR/IGT along with one of the following factors (HDL, TG, BMI), to maximize sensitivity.

As a sensitivity analysis, we created a second measure for MS from the International Diabetes Federation (IDF) consensus criteria^31^ which emphasizes obesity. The IDF criteria consists of central obesity, plus 2 of: raised TG (>1.7 mmol/l); reduced HDL (<1.03 mmol/l in males and <1.29 mmol/l in females); raised blood pressure (>130/85 mmHg); raised FPG (>5.6 mmol/l).

### Inflammatory Markers (Ages 9 and 18 y)

We examined IL-6 (age 9 y) and CRP (ages 9 and 18 y). IL-6 was not available at age 18 years. Blood samples were collected from non-fasting participants at age 9 years, and fasting participants at age 18 years.

### Measurement of Potential Confounders

Before analysis, we removed all participants with hsCRP >10,^16^ to address the potential confounder of ongoing/recent infection/inflammatory disease. We also omitted those with previous use of anti-psychotic medication due to the risk of reverse causation. For cross-sectional analyses at age 18 years, we adjusted for smoking (questionnaire data, coded categorically), BMI (clinic data, coded continuously), paternal social class at birth (questionnaire data, coded categorically), sex at birth (questionnaire data), ethnicity (questionnaire data, coded categorically), birthweight, and gestational age (clinic data, coded continuously). For analysis of MS, BMI was not adjusted for as this forms part of its diagnostic criteria. For our longitudinal analyses, we adjusted for BMI, paternal social class at birth, ethnicity, birthweight, and gestational age.

## Outcome Measures

### PEs (12 and 18 y)

PEs were identified through the face-to-face, semi-structured Psychosis-Like Symptom Interview (PLIKSi) conducted by trained psychology graduates in assessment clinics and were coded according to the definitions and rating rules for the Schedules for Clinical Assessment in Neuropsychiatry, Version 2.0.^32^ Inter-rater reliability for the age 12 PLIKSi was κ =0.75, with a test-retest reliability of κ = 0.48,^33^ suggesting “fair” agreement. At age 18 years, the PLIKSi has “good” inter-rater and test-retest reliability (both κ = 0.8).^34^ PEs covering the 3 main domains of positive psychotic symptoms occurring either in the preceding 6 months (measured at age 12 y), or since age 12 years (measured at age 18 y) were elicited: hallucinations (visual and auditory), delusions (spied on, persecuted, thoughts read, reference, control, grandiosity, and other), and thought interference (insertion, withdrawal, and broadcasting).

After cross-questioning, interviewers rated PEs as not present, suspected, or definitely present. For suspected or definite PEs, interviewers also recorded the frequency; affect, effects on social/educational/occupational function; help seeking; and attributions including fever, hypnopompic/hypnogogic state, or illicit drugs. Cases of PEs in this study were defined only as individuals with *definite* PEs to help confirm a relatively “high-risk” group for later development of clinical psychosis. The comparator group was suspected/no PEs.

### Psychotic Disorder (Age 18 y)

Psychotic disorder was defined by ALSPAC as the presence of PEs at least once per month in the preceding 6 months, when symptoms were not attributable to fever/sleep/drugs but caused significant distress and resulted in help-seeking from a professional source (general practitioner, counselor, mental health team), or significantly disrupted social/occupational function. This operational definition has been examined and used in previous work.^16,35^

## Statistical Analysis

A graphical display of the timeline of our included variables is outlined in table 1.

**Table 1.** Longitudinal Timeline of Included Variables

Age (y)	Exposure	Outcome	Mediator/ Moderator
9	IR,FPG, FI, IGT		IL-6, CRP
12		PEs	
18	IR, FPG, FI, MS	PEs, psychotic disorder	CRP

*Note*: IR, insulin resistance; PEs, Psychotic Experiences; IL-6, interleukin-6; CRP, C-reactive protein; IGT, impaired glucose tolerance; MS, Metabolic Syndrome; FI, fasting insulin.

### Cross-sectional and Longitudinal Relationships Between Dysglycaemia and PEs or Psychotic Disorder

Cross-sectional analyses examined the association between markers of dysglycaemia and PEs or psychotic disorder (age 18 y), while longitudinal analyses examined the association between markers of dysglycaemia (age 9 y) and PEs or psychotic disorder (ages 12 and 18 y). Biomarker values that were not normally distributed (all except FPG) were natural log-transformed. Resultant variables were standardized (Z-transformed) so the ORs represent the increase in risk of PE per SD increase in exposure.

ORs and 95% CI for PEs were estimated using logistic regression. Biomarkers were modeled as both continuous and binary independent variables (as per the clinical cut-off) in separate analyses. Quadratic terms were created separately for all continuous variables and entered into a logistic regression model to simulate curvilinear regression, to test the linearity of relationships between PEs and biomarkers.

### Cross-sectional and Longitudinal Relationships Between Dysglycaemia and Immune Activation

We completed univariable Pearson’s correlation analyses on metabolic and inflammatory biomarkers both cross-sectionally (age 18 y) and longitudinally (ages 9 and 18 y). We completed multivariable analyses with adjustments (as described above) on biomarkers showing evidence of an association.

### Mediation/Moderation Analyses Between Dysglycaemia, Inflammation and PEs/Psychotic Disorder

To test for moderation, we first completed a stratified analysis, in which the relationship between IL-6 (age 9 y) and PEs or psychotic disorder (age 18 y) in groups with and without IR separately was examined, and then, under a logistic model whether there was an interaction between IL-6 (age 9 y) and IR (ages 9 and 18 y) (IL-6xIR) on the outcome of PEs or psychotic disorder. To test for mediation, we used the PROCESS add-on for IBM SPSS 24.0 (available from http://www.afhayes.com). We tested for a significant indirect (mediation) effect for IR at age 18 years between IL-6 at age 9 years and PEs/psychotic disorder at age 18 years.

## Results

We included *n* = 2627 in our analyses. Table 2 presents clinical and biomarker characteristics of the sample at age 18 years. Our sample included *n* = 139 (5.2%) with definite PEs at age 12 years, *n* = 146 (5.4%) with definite PEs at age 18 years, and *n* = 37 (1.4%) with psychotic disorder at age 18 years.

**Table 2.** Characteristics of Sample at Age 18 y

Characteristic	All Sample	IR Absent^a^	IR Present^a^	Test Statistic^b^*P*-value
Participants, *n* (%)	2627 (100)	2417 (92)	210 (8)	—
Male sex, *n* (%)	1522 (49)	1403 (48.6)	119 (47.6)	0.10; *P* = .765
Father’s social class, *n* (%)				
I	143 (6)	136 (5.7)	7 (3.6)	9.27; *P* = .089
II	929 (36)	861 (36.3)	68 (35.1)	
III	1098 (43)	1018 (42.9)	80 (41.2)	
IV	335 (13)	305 (12.8)	30 (15.5)	
V	63 (2)	68 (2.3)	134 (4.6)	
White British ethnicity, *n* (%)	2574 (97)	2422 (97.9)	212 (97.7)	7.86; *P =* .453
BMI at 18 y, mean (SD), kg/m^2^	22.71	22.39 (3.42)	25.71 (5.56)	3.48 *P* < .001*
FPG at 18 y, mean (SD), mmol/l	5.03 (0.59)	5.00 (0.43)	5.34 (0.67)	3.49; *P* < .001*
HOMA_2_ at 18 y, mean (SD)	0.92 (0.73)	0.77 (0.32)	2.64 (1.52)	1.86; *P* < .001*
CRP at 18 y, mean (SD), mg/l	1.08 (1.41)	1.07 (1.40)	1.41 (1.62)	0.35; *P* < .001*
IL-6 at age 9 y, mean (SD), pg/ml	1.27 (1.59)	1.28 (1.62)	1.35 (1.41)	0.08; *P =* .531
CRP at age 9 y	0.68 (2.52)	0.69 (2.61)	0.68 (1.31)	0.01; *P =* .974
Birthweight, mean (SD), gram	3426.01 (549.86)	3431.02 (550.45)	3387.50 (525.38)	43.52; *P =* .249
Gestational age, mean (SD), wk	39.41 (1.84)	39.42 (1.83)	39.40 (1.70)	0.25; *P =* .841
Smoking at age 18 y, *n* (%) (current)	217 (6.86)	192 (6.6)	25 (10)	3.94; *P =* .043*

*Note*: BMI, body mass index; HOMA_2_, Homeostasis Model Assessment for Insulin Resistance; IL-6, interleukin-6; CRP, C-reactive protein.

^*^Denotes *P* < .05.

^a^HOMA_2_ score > 1.79.

^b^Categorical variables (sex, social class, ethnicity, smoking) were compared using a Chi-squared test, continuous variables (BMI, FPG, HOMA_2_, CRP, IL-6, Birthweight, Gestational Age) were compared using 2-tailed *t* test.

### Cross-sectional Association Between PEs and Markers of Dysglycaemia at Age 18

Using a categorical measure for IR, we found that IR was associated with PEs (adjusted OR = 2.32 [95% CI, 1.37–3.97] *P* < .001). Using the continuous measure for IR, HOMA_2_ demonstrated a nonlinear, exponential relationship with PEs (HOMA_2_ adjusted OR = 1.19 [1.01–1.43] *P* = 0.03; HOMA_2_ × HOMA_2_ adjusted OR = 1.08 [95% CI, 1.01–1.15] *P* = .02). FI demonstrated a linear relationship with PEs (adjusted OR = 1.23 [1.03–1.47] *P* = .02). FPG and MS were not associated with PEs (table 3). No associations were demonstrated using the psychotic disorder outcome (supplementary table 1).

**Table 3.** Cross-sectional Associations Between Metabolic Markers and PEs at Age 18

Predictor	OR (95% CI) for Psychotic Outcomes				
	Unadjusted Model			Adjusted for Sex, Ethnicity, Social Class, BMI^a^ and Smoking, Gestational Age, Birthweight	
Definite PEs	*n*	OR (95% CI)	*P*	OR (95% CI)	*P*
IR	2627	2.41(1.50–4.40)	<.001*	2.32 (1.37–3.97)	<.001*
HOMA_2_	2627	1.34 (1.17–3.18)	.019*	1.19 (1.01–1.43)	.034*
HOMA_2_ × HOMA_2_	2627	1.23 (1.06–3.09)	.008*	1.08 (1.01–1.15)	.021*
FPG	2627	0.89 (0.72–6.56)	.670	0.96 (0.13–7.28)	.966
FPG × FPG	2627	0.92 (0.82–1.06)	.762	0.99 (0.82–1.19)	.904
Fasting insulin	2627	1.36 (1.02–1.78)	.019*	1.23 (1.03–1.47)	.023*
Fasting insulin × fasting insulin	2627	1.07 (0.99–1.78)	.201	1.02 (0.95–1.10)	.526
Metabolic syndrome	2627	1.62 (0.77–3.39)	.204	1.68 (0.71–3.97)	.244

*Note*: BMI, body mass index; HOMA_2_, Homeostasis Model Assessment for Insulin Resistance; IR, insulin resistance; PEs, psychotic experiences. IR as categorical variable defined as HOMA_2_ >1.79. HOMA_2_, FPG, and Fasting Insulin presented as continuous variables.

^a^Not adjusted for in Metabolic Syndrome analysis.

*P < .05.

### Longitudinal Association Between Markers of Dysglycaemia (Age 9 y) and PEs (Ages 12 and 18 y)/ Psychotic Disorder (Age 18 y)

There were no longitudinal associations between markers of dysglycaemia at age 9 years and PEs at ages 12 or 18 years (table 4), or psychotic disorder (age 18 y) (supplementary table 2).

**Table 4.** Longitudinal Associations Between Metabolic Markers at Age 9 and PEs at Ages 12 and 18

Predictor	OR (95% CI) for Psychotic Outcomes				
	Unadjusted Model			Adjusted for Sex, Ethnicity, Social Class, BMI	
PE’s Age 12 y	*n*	OR (95% CI)	*P*	OR (95% CI)	*P*
2hrGT (age 9 y)	387	0.51 (0.26–9.99)	.048	0.77 (0.66–3.21)	.102
2hrGT × 2hrGT (age 9 y)	387	0.92 (0.71–1.20)	.527	0.91 (0.70–1.20)	.507
HOMA_2_ (age 9 y)	357	1.05 (0.66–1.68)	.712	1.10(0.79–1.53)	.559
HOMA_2_ × HOMA_2_ (age 9 y)	357	0.89 (0.11–7.01)	.913	0.84 (0.10–7.00)	.875
Fasting insulin (age 9 y)	357	1.14 (0.62–2.10)	.683	1.11 (0.70–1.91)	.721
Fasting insulin × fasting insulin (age 9 y)	357	0.85 (0.54–1.33)	.480	0.84 (0.54–1.31)	.447
FPG (age 9 y)	357	1.42 (0.51–4.00)	.503	1.56 (0.45–5.44)	.478
FPG × FPG (age 9 y)	357	0.42 (0.04–4.20)	.458	0.41 (0.04–4.18)	.451
IR (age 9 y)	357	1.25 (0.16,9.76)	.826	1.17 (0.14–9.80)	.881
IGT (age 9 y)	395	0.46 (0.17–1.21)	.122	0.72 (0.24–2.20)	.572
PE’s at age 18 y					
2hrGT (age 9 y)	337	1.08 (0.49–2.36)	.245	1.06 (0.48–2.33)	.890
2hrGT × 2hrGT (age 9 y)	337	0.62 (0.28–2.36)	.857	0.61 (0.27–1.48)	.229
HOMA_2_ (age 9 y)	352	1.05 (0.66–1.68)	.834	0.59 (0.28–1.25)	.171
HOMA_2_ × HOMA_2_ (age 9 y)	352	1.18 (0.66–2.03)	.754	1.12 (0.41–3.11)	.816
Fasting insulin (age 9 y)	352	0.66 (−0.33 to 1.23)	.178	0.73 (0.37–1.43)	.355
Fasting insulin × fasting insulin (age 9 y)	352	0.88 (0.48–1.63)	.684	0.88 (0.48–1.61)	.676
FPG (age 9 y)	352	-	-	-	-
FPG × FPG (age 9 y)	352	-	-	-	-
IR (age 9 y)	352	1.25 (0.16–9.76)	.699	-	-
IGT (age 9 y)	337	0.82 (0.27–2.50)	.734	0.93 (0.27–3.25)	.929

*Note*: 2hrGT, 2-hour glucose tolerance; BMI, body mass index; HOMA_2_, Homeostatic Measurement for Insulin Resistance; IR, insulin resistance; PEs, psychotic experiences; IGT, impaired glucose tolerance. IR and IGT as categorical variable defined as HOMA_2_ > 1.79 and IGT > 7.8 mmol. HOMA_2_, FPG, and Fasting Insulin presented as continuous variables.

- Unable to compute due to small *n.*

### Association Between IL-6, CRP, and Markers of Dysglycaemia

Cross-sectionally, CRP positively correlated with 2hrGT (age 9 y) (*P* < .01), HOMA_2_ (*P* < .01), and FI (*P* < .01) (age 18 y). Longitudinally, IL-6 (age 9 y) was positively correlated with HOMA_2_ (*P* < .01), FI (*P* < .01) and weakly negatively correlated with FPG (*P* < .01) (age 18 y). CRP (age 9 y) was positively correlated with HOMA_2_ (*P* < .01), FI (*P* < .01) and weakly negatively correlated with FPG (*P* = .03) (age 18 y; supplementary table 3). In multivariable analyses, evidence for a longitudinal association between serum IL-6 levels (age 9 y) and IR (age 18 y) remained after adjusting for potential confounders; adjusted OR = 1.65 (95% CI, 1.01–2.68) *P* = .04 (supplementary table 4).

### Mediation and Moderation Analyses Between Dysglycaemia, Inflammation and PEs/Psychotic Disorder

#### Results for Moderation Analysis

Three hundred twenty-six individuals (14%) met the criteria for IR at age 18 years. In univariable analyses, IL-6 (age 9 y) correlated with PEs (age 18) (*P* = .02). CRP (ages 9 and 18 y) did not correlate with PEs (age 18 y). Table 5 outlines the results of our stratification analysis. IL-6 was associated with PEs in the strata with IR (adjusted OR = 2.34 [95% CI, 1.02–5.33] *P* = .03), but not in the strata without IR (adjusted OR = 1.10 [95% CI, 0.84–1.45] *P* = .47). Our interaction analysis demonstrated an interaction between IL-6 (age 9 y) and IR (age 18 y) for PEs (age 18 y) (OR for interaction term = 2.18 [95% CI, 1.06–4.49] *P* = .03). There was no evidence for an interaction between IL-6 and IR (age 9 y) for PEs (age 18 y) (OR for interaction term = 1.29 [95% CI, 0.69–2.19] *P* = .49). Using the psychotic disorder outcome, IL-6 (age 9 y) was associated with psychotic disorder (age 18 y) in the strata without IR (adjusted OR = 1.63 [95% CI, 1.08–2.46] *P* = .02), but the effect was significantly stronger in the strata with IR (adjusted OR = 4.71 [95% CI, 1.38–16.07] *P* = .01) (OR for interaction term = 3.23 [95% CI, 1.08–11.65] *P* = .04; supplementary table 5).

**Table 5.** OR for IL-6 (Age 9 y) and PE (Age 18 y) Stratified by IR

	Odds Ratio (95% CI) for PEs at 18 yrs			Adjusted for Sex, Ethnicity, Social Class, BMI, Smoking, Gestational Age, Birthweight	
Group/Predictor	*n*	OR (SD)	*P*	OR (SD)	*P*
IR absent at 18 y					
IL-6 at age 9 y	2301	1.12 (0.83–1.43)	.338	1.10 (0.84–1.45)	.471
IR present at 18 y					
IL-6 at age 9 y	326	2.45 (1.24–4.84)	0.009*	2.34 (1.02–5.33)	.03*

*Note*: BMI, body mass index; IR, insulin resistance; PEs, Psychotic Experiences; IL-6, interleukin-6.

*P < .05.

#### Results for Mediation Analysis

There was no evidence of an indirect (mediation) effect, using IR (age 18 y) as the mediator variable between IL-6 (age 9 y) and PEs (age 18 y): coefficient <0.001; SE = 0.005; *P* = .982. Similar results were obtained using HOMA_2_ (age 18 y) as the potential mediator: coefficient = 0.01; SE = 0.01; *P* = .11. For the psychotic disorder outcome, there was no evidence of an indirect (mediation) effect using IR (age 18 y) as the mediator variable: coefficient = 0.01; SE = 0.01; *P* = .46.

#### Results for Sensitivity Analyses

The results tables for our sensitivity analyses for both the 75th centile-defined cut-off for IR, and for IDF-defined MS, are presented in the sensitivity analysis section of the supplementary data (supplementary tables 6–10). All associations demonstrated in our primary analyses were retained in the sensitivity analyses.

## Discussion

### Main Findings

In this secondary analysis of a large birth cohort, we examined whether dysglycaemia is associated with PEs/psychotic disorder at an earlier age than previous research has examined. We also investigated whether some of the known immune associations with psychosis may be part of a wider relationship involving dysglycaemia. Firstly, we hypothesized that dysglycaemia would be associated with PEs. After adjustments for confounders, we found that the continuous measure for HOMA_2_ was non- linearly (exponentially) associated with PEs, and the binary measure for clinical IR was even more strongly associated with PEs, at age 18 years. Additionally, the HOMA_2_ cut-off for our 75th centile-defined IR, which was lower (>1.50) than our primary cut-off (>1.79), demonstrated a less-strong association with PEs at age 18 years. Together, this provides face validity to our results, considering the non-linear (exponential) association between the continuous HOMA_2_ measure and PEs at age 18 years. There were no longitudinal associations between dysglycaemia at age 9 years and later PEs, or when using the outcome “psychotic disorder,” though the sample of participants at age 18 years with psychotic disorder was small thus the power of such analyses was limited.

A shared disease process may link dysglycaemia, immune activation and psychotic symptoms.^8,36^ Previous work using the same birth cohort has found that childhood IL-6 is longitudinally associated with PEs at age 18 years.^16^ We found that IL-6 correlates with markers of dysglycaemia longitudinally. We also found that, using both cut-offs for IR, there is a statistical interaction between IL-6 and IR for the outcome of both PEs and psychotic disorder at age 18 years. Interaction analyses, particularly of quantitative pattern as described here, are fraught with limitations and are historically difficult to replicate,^37^ thus our findings regarding moderation should be considered tentatively. Nonetheless, our results may indicate a shared pathophysiological process involving metabolic and inflammatory alterations in at least a subset of individuals who develop PEs, although extrapolating findings of statistical interaction to infer information about biological mechanisms is problematic.^38^ Biological plausibility may exist via a disruption in the PI3K/mTOR/AKT pathway, which is involved in insulin sensitivity, neuronal growth and axon elongation, dopamine regulation, and the innate and adaptive immune systems,^39–41^ and has previously been implicated as a potential mechanism linking schizophrenia and T2DM.^42^ Additionally, common risk factors for both psychosis and dysglycaemia include intra-uterine insults (trauma, infection, gestational diabetes) and early-life distress, both of which may involve inflammatory processes,^43,44^ contributing to an early-programming model of illness.

Our mediation analysis suggests that IR does not mediate the association between IL-6 and PEs/psychotic disorder. However, this mediation analysis was imperfect as we were unable to fit the mediator variable longitudinally in-between the exposure and outcome. We were also unable to test mediation reciprocally, ie, with IL-6 as a mediator between IR and PEs/psychotic disorder. Future work may seek to attempt to address these issues.

Interestingly, PEs were not associated with the binary variable of MS, either by WHO (dysglycaemia-centric) or IDF (obesity-centric) criteria. This finding may provide some evidence that the driver of metabolic abnormality in those with PEs, at least initially, is IR, and may foster increased emphasis on the use of WHO criteria for patients with psychosis. Several recent meta-analyses^8,45,46^ have shown a relationship between lipid metabolism and FEP. Our findings suggest that IR may be upstream of the wider MS, which would align with endocrine literature, that supports IR as an upstream driver of lipid dysfunction and other elements associated with chronic psychosis, such as cardiovascular disease and hypertension.^47^

We found no longitudinal associations between markers of dysglycaemia (age 9 y) and PEs (ages 12 and 18 y), after adjustments. These findings give weight to the hypothesis that immune dysfunction may be upstream of dysglycaemia in its relationship with psychosis.^48^ While a relatively smaller sample size for the longitudinal analysis may have resulted in type II error, another interpretation is that an abnormal metabolic process (measured by IR) that contributes to risk of PEs may be undeveloped by age 9 years.

### Strengths and Limitations

There are several strengths to our work. Firstly, to our knowledge this is the first study to investigate that metabolic abnormality might be present in those with PEs, and that it may be linked to immune activation. Secondly, the ALSPAC cohort provided the opportunity to conduct analyses on a relatively large and well characterized cohort and permitted longitudinal analyses. Thirdly, we were able to consider detailed potential confounders and minimize possible reverse causality secondary to the use of antipsychotic medication.

There are important limitations that should be taken into our account, meaning our results should be interpreted with caution. Firstly, IL-6 is but one cytokine in the constellation of immune activation. Future studies should include multiple pro and anti-inflammatory markers for a comprehensive understanding of the immune changes linking dysglycaemia and PEs. Additionally, the sample available for different analyses differed due to availability which may introduce a source of bias. Furthermore, since we included only those participants who received testing for all the variables we analyzed, selection bias may also be factor. We were also limited by the variables available for analysis; an ideal study would include a measure of IL-6 at age 18 years. Also, since a number of variables featured in the WHO classification^30^ for MS were unavailable, we aimed to maximize sensitivity potentially at the expense of specificity in our analysis. However, our sensitivity analysis using the IDF definition^31^ featured the whole criteria, and the results did not differ. In addition, clinical cut-offs for IR measured using HOMA_2_ may be problematic.^28^ We attempted to address this limitation by using the highest of the proposed cut-offs (to increase specificity), and to define a population-based cut-off using the 75th centile. Furthermore, while most biochemical tests were sampled in the fasting state, inflammatory markers at age 9 years were sampled in the non-fasting state, which may increase measurement error. Measurement error, if non-differential as is likely to be the case here, can introduce a bias towards the null, so the results for IL-6 may be underestimated. In addition, while longitudinal studies^34^ have shown PEs to be a robust estimator for a later diagnosis of psychotic illness, we did not examine a clinical sample with diagnosed schizophrenia. We attempted to manage this limitation by including the psychotic disorder outcome as a secondary analysis, though the power of such analyses were limited. Interestingly, depression has also shown significant associations with childhood immune processes^16^ and dysglycaemia.^48^ Future research should longitudinally examine the association and direction of association between inflammation, dysglycaemia, and depression. Finally, while we were able to adjust for multiple possible confounders in our analysis, we were unable to account for the possible contribution of perceived stress and chronic activation of the hypothalamic-pituitary axis in our findings. Future research may seek to address this.

### Implications and Future Directions

Our results provide evidence that even before the onset of clinical psychosis, some patients may have an active process involving IR and inflammation, which may predispose to further metabolic dysfunction. It is possible that inflammation and metabolic risk factors interact to increase risk of psychosis in some people. That IR is associated with PE’s even after controlling for potential confounding factors is important and may provide evidence for clinicians and those involved in clinical guideline formation, to the potential need for more sensitive examination of baseline metabolic function at FEP. As some second-generation antipsychotics are diabetogenic, careful selection of medication accounting for sensitive metabolic dysfunction may reduce the risk of iatrogenic “toppling” into clinical type-2 diabetes.

## Funding

B.I.P. is supported by an Academic Clinical Fellowship from the National Institute for Health Research. G.M.K. is supported by an Intermediate Clinical Fellowship from the Wellcome Trust (201486/Z/16/Z) and a Clinical Lecturer Starter Grant from the Academy of Medical Sciences, UK (80354). S.Z. is supported by the NIHR Bristol Biomedical Research Centre. Funding for data access was obtained from Coventry and Warwickshire Partnership NHS Trust R&I Department, from the “Research Development Fund.” The UK Medical Research Council and Wellcome Trust (102215/2/13/2) and the University of Bristol provide core support for ALSPAC.

## Supplementary Material

Supplementary Material
