# Supplementary material for: Dysglycaemia, Inflammation and Psychosis: Findings From the UK ALSPAC Birth Cohort
Source: Schizophr Bull. 2018 Apr 9;45(2):330–8. doi: 10.1093/schbul/sby040 (PMC6403055; doi:10.1093/schbul/sby040)
Supplement: Supplementary Material [file sby040_suppl_supplementary_materials.doc]

**Dysglycaemia, inflammation and psychosis: findings from the U.K. ALSPAC birth cohort***Perry, B.I.; Upthegrove, R.; Thompson, A.; Marwaha, S.; Zammit, S.; Singh, S.P.; Khandaker, G.M*

Online Supplementary Material

*Supplementary Data Table 1: Cross-sectional associations between dysglycaemia and psychotic disorder (age 18 years)*

| **Predictor** | **Odds Ratio (95% C.I.) for Psychotic Outcomes** | | | | |
| --- | --- | --- | --- | --- | --- |
|  | **Unadjusted Model** | | | **Adjusted for sex, ethnicity, social class, BMI^1^ and smoking, gestational age, birthweight** | |
| **Psychotic Disorder** | **n** | **OR(95% C.I.)** | **p** | **OR(95% C.I.)** | **p** |
| Insulin Resistance | 2627 | 1.73 (0.73-4.11) | 0.218 | 1.63 (0.60-4.45) | 0.598 |
| Insulin Resistance (75% centile) | 2627 | 1.65 (0.66-3.89) | 0.325 | 1.45 (0.59-4.01) | 0.458 |
| HOMA_2_ | 2627 | 1.11 (0.82-1.49) | 0.505 | 1.16 (0.80-1.68) | 0.998 |
| HOMA_2_*HOMA_2_ | 2627 | 1.02 (0.88-1.17) | 0.810 | 1.00 (0.85-1.17) | 0.982 |
| FPG | 2627 | 0.89 (0.61-1.30) | 0.550 | 0.80 (0.52-1.28) | 0.303 |
| FPG*FPG | 2627 | 0.92 (0.72-1.17) | 0.501 | 0.91 (0.68-1.21) | 0.509 |
| Fasting Insulin | 2627 | 1.10 (0.82-1.48) | 0.536 | 1.15 (0.79-1.67) | 0.458 |
| Fasting Insulin*Fasting Insulin | 2627 | 1.02 (0.90-1.15) | 0.764 | 1.00 (0.79-1.67) | 0.960 |
| Metabolic Syndrome | 2627 | 1.84 (0.56-6.03) | 0.312 | 1.11 (0.25-5.00) | 0.896 |
| Metabolic Syndrome (IDF criteria) | 2627 | 1.65 (0.45-6.54) | 0.691 | 1.19 (0.56-5.67) | 0.591 |

*Supplementary Data Table 2: Longitudinal Associations between dysglycaemia (age 9 years) and psychotic disorder (age 18 years)*

| **Predictor** | **Odds Ratio (95% C.I.) for Psychotic Outcomes** | | | | |
| --- | --- | --- | --- | --- | --- |
|  | **Unadjusted Model** | | | **Adjusted for sex, ethnicity, social class, BMI** | |
| **Psychotic Disorder at age 18** | **n** | **OR(95% C.I.)** | **p** | **OR(95% C.I.)** | **p** |
| 2hrGT (age 9) | 337 | 1.17 (0.07-20.95) | 0.918 | 1.21 (0.07-22.82) | 0.896 |
| 2HrGT*2hrGT (age 9) | 337 | 0.07 (0.00-11.19) | 0.298 | 0.07 (0.00-10.99) | 0.302 |
| HOMA_2_ (age 9) | 352 | 0.48 (0.17-1.30) | 0.148 | 0.57 (0.20-1.64) | 0.297 |
| HOMA_2_*HOMA_2_ (age 9) | 352 | 1.36 (0.74-2.50) | 0.325 | 1.38 (0.74-1.64) | 0.572 |
| Fasting Insulin (age 9) | 352 | 0.44 (0.14-1.35) | 0.141 | 0.51 (0.16-1.66) | 0.265 |
| Fasting Insulin*Fasting Insulin (age 9) | 352 | 1.38 (0.74-2.59) | 0.313 | 1.39 (0.76-2.63) | 0.311 |
| FPG (age 9) | 352 | 0.78 (0.28-2.55) | 0.678 | 0.91 (0.27-3.10) | 0.878 |
| FPG*FPG (age 9) | 352 | 0.96 (0.52-1.77) | 0.898 | 0.96 (0.27-3.07) | 0.883 |
| Insulin Resistance (age 9) | 352 | 1 | 1 | 1 | 1 |
| Insulin Resistance (age 9) (75^th^ centile) | 352 | 1 | 1 | 1 | 1 |
| IGT (age 9) | 337 | 1.42 (0.13-15.86) | 0.773 | 1.50 (0.13-16.96) | 0.743 |
| ^1^*n* too small for analysis | | | | | |

|  | | FPG @ age 18 years | HOMA_2_ @ age 18 years | Fasting Insulin @age 18 years | CRP @ age 18 years | FPG @ age 9 years | 2hrGT @ age 9 years | HOMA_2_ @ age 9 years | Fasting Insulin @ age 9 years | Cortisol @ age 9 years | IL-6 @ age 9 years | CRP @ age 9 years |
| --- | --- | --- | --- | --- | --- | --- | --- | --- | --- | --- | --- | --- |
| FPG @ age 18 years | r (p) | 1 | .309^**^ (<0.001) | .263^**^ (<0.001) | -.034 (0.34) | .245^**^(<0.01) | -.012 (0.81) | -.006 (0.91) | -.015 (0.76) | -.048 (0.32) | -.045^*^ (0.03) | -.046^*^(0.03) |
|  | N | 3208 | 3138 | 3141 | 3208 | 437 | 418 | 437 | 437 | 435 | 2176 | 2180 |
| HOMA_2_ @ age 18 years | r (p) | .309^**^(<0.001) | 1 | .995^**^ (<0.001) | .132^**^(<0.001) | .032 (0.51) | .027 (0.59) | .147^**^ (0.002) | .145^**^(0.003) | -.016 (0.74) | .068^**^(0.002) | .082^**^(<0.001) |
|  | N | 3138 | 3138 | 3138 | 3138 | 428 | 410 | 428 | 428 | 426 | 2125 | 2129 |
| Fasting Insulin @ age 18 years | r (p) | .263^**^ (<0.001) | .995^**^ (<0.001) | 1 | .139^**^(<0.001) | .025 (0.60) | .029 (0.56) | .159^**^ (<0.001) | .157^**^ (0.001) | -.019 (0.70) | .069^**^(0.001) | .087^**^(<0.001) |
|  | N | 3141 | 3138 | 3141 | 3141 | 428 | 410 | 428 | 428 | 426 | 2127 | 2131 |
| CRP @ age 18 years | r (p) | -.034 (0.06) | .132^**^(<0.001) | .139^**^(<0.001) | 1 | .030 (0.53) | .017 (0.72) | .085 (0.08) | .082 (0.09) | .036 (0.45) | .076^**^(<0.001) | .294^**^(<0.001) |
|  | r (p) | 3208 | 3138 | 3141 | 3208 | 437 | 418 | 437 | 437 | 435 | 2176 | 2180 |
| FPG @ age 9 years | r (p) | .245^**^(<0.001) | .032 (0.51) | .025 (0.60) | .030 (0.53) | 1 | -.030 (0.55) | .139^**^(0.004) | .112^*^(0.01) | .029 (0.56) | -.056 (0.31) | -.060 (0.27) |
|  | N | 437 | 428 | 428 | 437 | 437 | 408 | 437 | 437 | 434 | 335 | 335 |
| 2hr GT @ age 9 years | r (p) | -.012 (0.86) | .027 (0.59) | .029 (0.56) | .017 (0.72) | -.030 (0.55) | 1 | -.025 (0.62) | -.019 (0.70) | .114^*^ (0.02) | .053 (0.34) | .149^**^ (0.01) |
|  | N | 418 | 410 | 410 | 418 | 408 | 418 | 408 | 408 | 407 | 325 | 325 |
| HOMA_2_ @ age 9 years | r (p) | -.006 (0.91) | .147^**^ (0.002) | .159^**^ (0.01) | .085 (0.08) | .139^**^ (0.004) | -.025 (0.62) | 1 | .994^**^(<0.001) | .032 (0.51) | .060 (0.27) | .095 (0.08) |
|  | N | 437 | 428 | 428 | 437 | 437 | 408 | 437 | 437 | 434 | 335 | 335 |
| Fasting Insulin @ age 9 years | r (p) | -.015 (0.76) | .145^**^ (0.003) | .157^**^ (0.001) | .082 (0.09) | .112^*^ (0.02) | -.019 (0.70) | .994^**^(<0.001) | 1 | .028 (0.56) | .064 (0.24) | .102 (0.06) |
|  | N | 437 | 428 | 428 | 437 | 437 | 408 | 437 | 437 | 434 | 335 | 335 |
| Cortisol @ age 9 years | r (p) | -.048 (0.32) | -.016 (0.74) | -.019 (0.70) | .036 (0.45) | .029 (0.56) | .114^*^ (0.02) | .032 (0.51) | .028 (0.56) | 1 | .027 (0.03) | .058 (0.30) |
|  | N | 435 | 426 | 426 | 435 | 434 | 407 | 434 | 434 | 435 | 333 | 333 |
| IL-6 @ age 9 years | r (p) | -.045^*^ (0.04) | .068^**^ (0.002) | .069^**^ (0.001) | .076^**^(<0.001) | -.056 (0.31) | .053 (0.34) | .060 (0.27) | .064 (0.24) | .027 (0.63) | 1 | .451^**^(<0.001) |
|  | N | 2176 | 2125 | 2127 | 2176 | 335 | 325 | 335 | 335 | 333 | 2176 | 2176 |
| CRP @ age 9 years | r (p) | -.046^*^ (0.03) | .082^**^(<0.001) | .087^**^(<0.001) | .294^**^(<0.001) | -.060 (0.27) | .149^**^ (0.007) | .095 (0.08) | .102 (0.06) | .058 (0.29) | .451^**^(<0.001) | 1 |
|  | N | 2180 | 2129 | 2131 | 2180 | 335 | 325 | 335 | 335 | 333 | 2176 | 2180 |
| *denotes significance at p<0.05  *denotes significance at p<0.01  Cross-sectional  Longitudinal | | | | | | | | | | | |  |

*Supplementary Data Table 3: Biomarker Correlations (Pearson’s Correlation)*

| **Predictor** | **Odds Ratio (95% CI) for Insulin Resistance per SD increase in IL-6/ CRP at 9 years** | | | | |
| --- | --- | --- | --- | --- | --- |
|  | **Unadjusted Model** | | | **Adjusted for sex, ethnicity, social class, BMI and smoking, gestational age, birthweight** | |
| **IR age 9** | **n** | **OR(SD)** | **p** | **OR(SD)** | **p** |
| IL-6 | 335 | 1.24 (0.85-1.83) | 0.267 | 1.15 (0.72-1.84) | 0.556 |
| CRP | 335 | 1.40 (0.97-2.01) | 0.073 | 1.00 (0.59-1.71) | 0.903 |
| **IR age 18** |  |  |  |  |  |
| IL-6 (age 9) | 2126 | 1.67 (1.09-2.56) | 0.024 | 1.65 (1.01-2.68) | 0.037* |
| CRP (age 9) | 2130 | 1.21 (1.03-1.43) | 0.023 | 0.84 (0.67-1.06) | 0.114 |
| CRP (age 18) | 3140 | 1.89 (1.43-2.50) | <0.001* | 1.18 (0.81-1.74) | 0.389 |
| *denotes p<0.05 | | | | | |

*Supplementary Data Table 4: Longitudinal and cross-sectional associations between inflammation and IR*

| **Group/Predictor** | **Odds Ratio (95% CI) for Psychotic Disorder at 18** | | | **Adjusted for sex, ethnicity, social class, BMI, smoking, gestational age, birthweight** | |
| --- | --- | --- | --- | --- | --- |
|  | **n** | **OR (SD)** | **p** | **OR (SD)** | **p** |
| **IR absent at 18y** |  |  |  |  |  |
| IL-6 at age 9 | 2301 | 1.51 (1.02-2.22) | 0.038* | 1.63 (1.08-2.46) | 0.019* |
| **IR present at 18y** |  |  |  |  |  |
| IL-6 at age 9 | 326 | 4.88 (1.44-16.54) | 0.011* | 4.71 (1.38-16.07) | 0.013* |
| *denotes p<0.05 | | | | | |

Supplementary Data Table 5: Stratification of IR on association between IL-6 and psychotic disorder (ages 9 and 18 years)

**Sensitivity Analysis 1: 75^th^ Centile for clinical IR**

*Supplementary Data Table 6: Cross sectional analysi*s – IR and PEs at age 18 years

| **Predictor** | **Odds Ratio (95% C.I.) for Psychotic Outcomes** | | | | |
| --- | --- | --- | --- | --- | --- |
|  | **Unadjusted Model** | | | **Adjusted for sex, ethnicity, social class, BMI^1^ and smoking, gestational age, birthweight** | |
| **Definite PEs (age 18)** | **n** | **OR(95% C.I.)** | **p** | **OR(95% C.I.)** | **p** |
| Insulin Resistance (75^th^ centile) (age 18) | 2627 | 2.14 (1.40-3.27) | <0.001* | 1.82 (1.11-2.99) | 0.019* |
| *denotes p<0.05 | | | | | |

*Supplementary Data Table 7:* Longitudinal analysis – IR (ages 9 & 18 years) and PEs (ages 12 & 18 years)

| **Predictor** | **Odds Ratio (95% C.I.) for Psychotic Outcomes** | | | | |
| --- | --- | --- | --- | --- | --- |
|  | **Unadjusted Model** | | | **Adjusted for sex, ethnicity, social class, BMI** | |
| **PEs age 12** | **n** | **OR(95% C.I.)** | **p** | **OR(95% C.I.)** | **p** |
| Insulin Resistance (75^th^ centile) (age 9) | 357 | 0.92 (0.21-4.14) | 0.917 | 1.10 (0.24-5.10) | 0.906 |
| **PEs at age 18** |  |  |  |  |  |
| Insulin Resistance (75^th^ centile) (age 9) | 352 | **1** |  | **1** |  |
| *unable to compute due to small *n* | | | | | |

*Supplementary Data Table 8:* IL-6 and PEs stratified by IR

| **Group/Predictor** | **Odds Ratio (95% CI) for PEs at 18** | | | **Adjusted for sex, ethnicity, social class, BMI, smoking, gestational age, birthweight** | |
| --- | --- | --- | --- | --- | --- |
|  | **n** | **OR (SD)** | **p** | **OR (SD)** | **p** |
| **IR (75^th^ centile) absent at 18y** |  |  |  |  |  |
| IL-6 at age 9 | 2301 | 1.04 (0.65-1.32) | 0.235 | 1.10 (0.84-1.45) | 0.474 |
| **IR (75^th^ centile) present at 18y** |  |  |  |  |  |
| IL-6 at age 9 | 326 | 2.14 (1.20-3.99) | 0.002* | 2.09 (1.01-4.56) | 0.031* |
| *denotes p<0.05 | | | | | |

*Supplementary Data Table 9:* Interaction analysis – IL-6xIR and PEs

| **Predictor** | **Odds Ratio (95% C.I.) for Psychotic Outcomes** | | | | |
| --- | --- | --- | --- | --- | --- |
|  | **Unadjusted Model** | | | **Adjusted for sex, ethnicity, social class, BMI^1^ and smoking, gestational age, birthweight** | |
|  |  | | |  | |
| **Definite PEs (age 18)** | **n** | **OR(95% C.I.)** | **p** | **OR(95% C.I.)** | **p** |
| IL-6*IR(75^th^ centile) (age 18) | 2014 | 2.20 (1.23-4.27) | 0.012* | 2.08 (1.04-2.99) | 0.041* |
| IL-6*IR(75^th^ centile) (age 9) | 314 | 0.87 (0.34-8.34) | 0.456 | 0.81 (0.23-6.23) | 0.765 |
| *denotes p<0.05 | | | | | |

**Sensitivity Analysis: MS as defined by IDF Criteria**

*Supplementary Data Table 10:* Cross sectional analysis – MS and PEs at age 18 years

| **Predictor** | **Odds Ratio (95% C.I.) for Psychotic Outcomes** | | | | |
| --- | --- | --- | --- | --- | --- |
|  | **Unadjusted Model** | | | **Adjusted for sex, ethnicity, social class, BMI^1^ and smoking, gestational age, birthweight** | |
| **Definite PEs (age 18)** | **n** | **OR(95% C.I.)** | **p** | **OR(95% C.I.)** | **p** |
| Metabolic Syndrome (IDF Criteria) | 2627 | 1.45 (0.86-2.18) | 0.547 | 1.23 (0.87 -1.45) | 0.635 |
